# The Irrationality of GOF Avian Influenza Virus Research

**DOI:** 10.3389/fpubh.2014.00077

**Published:** 2014-07-16

**Authors:** Simon Wain-Hobson

**Affiliations:** ^1^Molecular Retrovirology Unit, Department of Virology, Institut Pasteur, Paris, France; ^2^Foundation for Vaccine Research, Washington, DC, USA

**Keywords:** avian influenza, airborne transmission, human adaptation, DURC, unfalsifiable

## Abstract

The last two and a half years have witnessed a curious debate in virology characterized by a remarkable lack of discussion. It goes by the misleading epithet “gain of function” (GOF) influenza virus research, or simply GOF. As will be seen, there is nothing good to be gained. The controversial experiments confer aerosol transmission on avian influenza virus strains that can infect humans, but which are not naturally transmitted between humans. Some of the newer strains are clearly highly pathogenic for man. It will be shown here that the benefits of the work are erroneous and overstated while the risk of an accident is finite, if small. The consequence of any accident would be anywhere from a handful of infections to a catastrophic pandemic. There has been a single open international meeting in this period, which is surprising given that openness and discussion are essential to good science. Despite US and EU government funding, no risk–benefit analysis has been published, which again is surprising. This research can be duplicated readily in many labs and requires little high tech. It falls under the definition of DURC without the slightest shadow of a doubt and constitutes the most important challenge facing contemporary biology.

Science excels in making things that work: vaccines, smart phones, and airplanes. This is the implicit promise made to society and one underpinned by basic science. “If you invest in the biomedical research enterprise, ultimately it will deliver products that will impact global health and alleviate suffering.” Biomedicine has delivered on this promise with spectacular success – average life expectancy is now out to 80 years and beyond in some countries.

Making things that work relies on solid data that resist the tests of time. With a background in HIV evolution and genetics, I became drawn to the latest hot topic in virology, which is to predict the future of rapidly evolving viruses such as avian influenza A H5N1 or H7N9 (1–4). I was bothered because the claims about delivering vaccines and drugs based on lab experiments did not square with my understanding of rapidly mutating viruses (5).

The issue is how these avian viruses will evolve and whether we can anticipate their trajectories by performing accelerated or forced evolution experiments in the lab. If yes, the reasoning goes that we can make vaccines out of these strains, develop drugs, and stockpile them, so heading off a future pandemic, assuming that a similar, but this time naturally arising strain, emerged. The vaccine goal is the most touted of benefits, no doubt because of their phenomenal cost–benefit ratio.

In the last 100 years, influenza pandemics occurred in 1918, 1957, 1968, 1977, and 2009, meaning that a pandemic can strike every 10–40 years. Influenza A viruses are distinguished by one of 16 hemagglutinins (H) and one of 9 neuraminidase (N) proteins on the surface of the virus, essentially marking them out antigenically. There are 110 strains of avian influenza viruses carrying one of 16 hemagglutinins (H1–H16) and one of 9 neuraminidase (N1–N9) proteins. The reservoir of influenza viruses in ducks, shorebirds, birds, and chickens is by far the largest among animals, although it is also found among pigs, dogs, and horses, to mention a few species.

As viral crystal-ball-gazing is a new topic with a scant literature, let us look at the track record for predicting influenza pandemics. Pandemic viruses in man are referred to as H1N1 (1918, 1977, and 2009), H2N2 (1957), and H3N2 (1968). For the Spanish flu virus (H1N1 1918), all eight segments of the genome came from bird strains. The 1957 H2N2 and 1968 H3N2 viruses represented mixes of more avian viruses along with parts of the 1918 virus. 1977 H1N1 represented an accidental reintroduction of an old vaccine strain pre-1957, probably from a Russian research lab (6).

Only by the twenty-first century did we have the wherewithal in evolutionary and genetic terms to have even the slightest chance of predicting a pandemic. All bets were on an avian virus spilling over in SE Asia. The 2009 H1N1 pandemic was a surprise on at least three counts: (1) it emerged from swine, (2) it was in NW Mexico, and (3) it represented the first time that a pandemic was initiated by a strain belonging to a virus that was already circulating – descendants of 1977 H1N1 were around in 2009 (7). In short, flu virologists were far from the mark (8–10).

Other avian influenza viruses cross over to humans causing mild to severe disease with case fatality rates that can sometimes approach 60%. Despite this, these viruses are hardly ever transmitted between humans because they lack the mutations enabling them to grow well in the upper respiratory tract. As such, they represent dead-end infections. In the last 17 years, the number of unequivocally documented H5N1 cases in humans is of the order of 650 or so. For H7N9 human infections, the number is 448 and rising. For both examples, the viruses spilled over directly from mixtures of duck, chicken, and bird strains. H7N9 was totally off the radar when it first struck in China in 2013 (11). Indeed, there had not been a single prior report of H7N9 in humans, which was very worrying and again illustrates how little we still know about flu viruses. However, when turning to serological surveys, which are woefully few, one finds that viruses from at least 11 hemagglutinin groups can be detected in farm workers in China (12). Presumably, these represent mild to asymptomatic infections that essentially go unrecorded. It shows that very probably large numbers of avian influenza viruses silently spill over to humans without any fuss.

In an attempt to recapitulate the evolutionary process and to anticipate the future, two groups performed forced evolution experiments on avian H5N1 influenza strains from 2004 and 2005. They used a ferret transmission model. The ferret is the animal of choice in influenza research for a number of reasons, one of which is that the animal sneezes, much as humans do. When housed in adjacent cages with an airflow carrying aerosols from the infected animal across to the receiver ferret, it is possible to ascertain whether a virus is capable of efficient airborne transmission. By repeating the process four to five times, they rapidly selected for such viruses (1, 2). Actually, the number of ferrets in each experiment was so small as to invite criticism on statistical grounds alone (13). While the ferret model has its limits, as pandemic human viruses are transmissible between ferrets by the airborne route, it could be assumed that these viruses will be so.

A more recent study on an H7N1 strain started with a virus that was lethal in ferrets with neurological complications (4). With minimal effort, a strain transmitted by the airborne route was obtained without loss of pathogenicity. In the earlier studies on H5N1 avian influenza, few ferrets showed respiratory distress. Now we are dealing with strains capable of a lethal respiratory-acquired infection. Obviously the “proof” experiment, inoculation of human volunteers with one of these lab-generated viruses, cannot be ethically performed. Yet, this creates a very unsatisfactory situation because science is about resolving conjecture, not making it. Assessing the risk to humans is equally stymied by these unfalsifiable findings, to use a Popperian term. Needless to say, precautionary logic and a savant interpretation of Murphy’s Law suggests that these viruses should be considered as highly dangerous for man. In short, the risk level has been enhanced by this work.

At a 2014 meeting on infectious diseases, Dr. Kawaoka reported experiments whereby he forced the evolution of the pandemic H1N1 2009 virus so that it could escape from natural human antibody responses. The experiment was not complicated: it involved simply mixing virus with sera from individuals who had been naturally infected and selecting out the virus that was not neutralized. A total of 15 sites on the virus hemagglutinin protein were identified. Concentrating on five of these sites, he was able to produce strains that completely escaped human antibodies and extant vaccine coverage. This experiment is different from prior avian influenza virus gain of function (GOF) experiments in that we know the virus is readily transmissible among humans – after all these lab-made strains are derived from the pandemic H1N1 2009 virus! As the strains escape vaccine control, they constitute, unambiguously, a HUGE risk to man.

Increased risk *per se* should not be frowned on if there are substantial benefits to be had. So what are the purported benefits of influenza A GOF research?

As the proponents talk about stockpiling preventive vaccines (4), we will examine this hypothesis. Given the annual change in the antigenic composition of a virus, the tried and tested working rule toward making a vaccine is to select from circulating strains those that are most likely to cover the world’s population in the next few months. The CDC has just reiterated this logic by their choice of viruses for the season 2014–15 (14). A single antigenic mismatch can substantially reduce vaccine efficiency. As mentioned above, there are 110 genetically confirmed combinations of avian hemagglutinin and neuraminidases presently circulating in ducks, shorebirds, and birds.

Perhaps a subset of these 110 avian viruses might pose a threat to man, yet we simply do not know this. The recent isolation of H10N8 from two patients, one of who had underlying immunosuppression and subsequently died (15), sparked three commentaries from influenza virologists along the lines of “H10N8, the next pandemic?” (16–18). Such dramatic extrapolation from two case reports does not help for sound science. But if we take them at face value, then we need a vaccine to this strain.

For complete coverage in the US, the cost of 314 million doses of a commercially available influenza vaccine to the public sector is presently between $6 and 15 per dose^1^, or between $1.9 and 4.7 billion. To be fully prepared for a pandemic would require preventive and stockpiled vaccines for all 110 strains. This ramps up the cost to something of the order of $209–517 billion. As the shelf life of an inactivated vaccine is ~12 months at 2–8°C, these would be annual costs^2^. Even if the number of vaccines were reduced to one per hemagglutinin (there are more distinct H than N proteins), a minimum number would be 16 stockpiled vaccines, or $30–75 billion annual costs.

These back-of-an-envelope calculations show that stockpiling vaccines is effectively science fiction, even if, with economies of scale, costs could be slashed by a factor of 10. Of course, these numbers ignore investments in staff and production facilities, to mention just a couple of issues. By comparison, radical investment in developing a near-universal flu vaccine, or vaccines that induced broad immune responses might be much cheaper.

Regarding the development of anti-viral drugs, the response is binary. If the virus is sensitive, society will go with what already exists. If not, the development of novel drugs normally involves a >10-year-cycle to get to market with a winnowing down of a large number of candidate molecules down to a very small number. Again the costs are huge and without a clear virus strain in the crosshairs, backed up by a scientific consensus, industry will not rise to the challenge.

What is the chance of experimentally settling on a combination of mutations that allows, say, avian H12N5 to become transmissible between ferrets that could also be thrown up by nature? I, for one, have not the slightest idea. You could perhaps document a restricted number of mutations but these would still be a reasonably large number allowing numerous permutations. The notion of hitting on a single solution is highly erroneous and constitutes a flawed appreciation of evolution. For example, over the course of evolution, the eye evolved independently something like 40 times. Taking an example from my world of retroviruses, there are at least six different ways to express the reverse transcriptase gene.

And so it goes with influenza. While the initial papers on H5N1 showed that the hemagglutinin gene had acquired important mutations to allow it to bind to human receptor molecules in the upper respiratory tract, a subsequent paper showed that the same mutations did not confer the same phenotype on other H5N1 viruses currently circulating (19). This is not surprising to virologists, or scientists with knowledge of protein structure. The overriding question is how many solutions are out there? It may not be possible to answer this accurately, although with techniques such as saturation mutagenesis, it may be possible to asymptote toward defining a fraction for an individual flu protein. With regular exchange of avian influenza genes, it will be an extraordinarily difficult challenge.

To resume, virological crystal-ball-gazing is even harder than the real thing. Virology can only deliver a limited number of answers in this area, very few of which may be useful for the development of effective pandemic vaccines, anti-viral drugs, or enhanced pandemic preparedness. By contrast, rapid surveillance and communication of findings seems to be *de rigueur* and have been shown to work in the real world. Existing networks are picking up isolated cases of H6N1 and H10N8 avian influenza meaning that they are doing a very good job (15, 20). In all likelihood, these will be dead-end infections that will not set off a pandemic, which are rare events given past information.

These forced evolution influenza virus experiments are most unlikely to deliver much practical information, nothing that a Health Minister could mobilize around. Meanwhile the risks are finite and small, but of catastrophic proportions if ever there was a breakdown of biosafety or biosecurity.

Advocates of this GOF research are off the mark for three other reasons. First, scientists are notoriously optimistic about their work and systematically underestimate risk (21). When pushed, they can hardly find a web page or reference citing the data about lab accidents. Second, they feel that once funded they should be free to publish – it has become a struggle for many in what is a crazily competitive race where publishing in big journals is a question of survival. “A paper in Nature is worth every risk” is how a colleague who fled Budapest in 1956 summed it up. Certainly, scientists should be as unfettered as possible, but enhancing the danger level of a virus impacts public safety and society as a whole. Society is the ultimate arbiter, a fact revealed by the scientists themselves in their grant proposals and papers where they explain how dangerous their virus is. They never miss an occasion that includes mortality statistics in a basic science manuscript, or to point out that a vaccine and drugs to their virus are lacking. *So why is it they are so refractory to discussion and openness?* Where are the three or four flu congresses where this topic has been openly debated?

This inability to discuss goes further. Surprisingly, no government agency, learned body, or independent organization has commissioned a comprehensive risk–benefit analysis on GOF influenza research, despite more than 2 years of controversy. Almost none of my colleagues are aware of the 2007 InterAcademy Panel (IAP) statement that “Scientists [too] have an obligation to do no harm.”^3^. Indeed, let us discuss what is and what is not DURC, but let us not hide or forget the IAP statement.

The third point concerns dissemination of information. These flu virologists are not trying to hurt their fellow beings, nor deliberately trying to set off an influenza pandemic. Yet, they are part of the paper race to publish. Once published, these studies can be reproduced at far less cost. Knowledge that it can be done is enough. And if reproduced in other labs with lower containment facilities or scrutiny, these strains could well proliferate, the corollary being an increased risk of an accidental release. Most scientists would see this as not part of their brief; “I did my bit, I’m not responsible for others.” In short, the larger picture, that of DURC, is simply not on their radar, the terms not part of their vocabulary. This absence of reflection was apparent in the debate about redacting parts of the original manuscripts on H5N1 GOF research. The papers were uploaded via the Internet, which means that they were on the cloud. Any computer security jock could find the data. Correct? I asked a colleague to track down a manuscript from my lab that had uploaded to a major journal. He had only the title page and agreed not to hack the Institut Pasteur server, which would have been too easy. He retrieved a complete pdf of the manuscript compiled by the journal web site in <2 min! This shows just how computer naïve the original discussion was.

The DURC issue, particularly in our Internet age, needs far more debate. Even though the risk of an error or a lab accident may be small, the consequences could be catastrophic. For this work to proceed, there needs to be a clear consensus based on an open discussion that the benefits outweigh the risks. Judging by the controversy, a consensus is clearly lacking (22, 23).

By resorting to semantics, or hiding behind the cloak of freedom to investigate, or whipping up fears about increased regulation, yet continuing GOF work, these researchers are showing themselves to be remarkably cavalier, disdainful of public opinion, and totally averse to discussion, which is a contradiction in terms for scientists. Their imperviousness will ultimately boomerang on the flu community that has shown an esprit de corps typical of a medieval guild. Ultimately, society will have the last word. My fear is that before the issue is settled there will be an accident or an incident resulting in a terrible backlash on biomedicine. Thereafter, the new dynamics will harsher. I do not even want to contemplate the nightmare of a man-made pandemic.

## Conflict of Interest Statement

The author declares that the research was conducted in the absence of any commercial or financial relationships that could be construed as a potential conflict of interest.

## Footnotes

^1^http://www.cdc.gov/vaccines/programs/vfc/awardees/vaccine-management/price-list/#adflu

^2^http://www.who.int/immunization_standards/vaccine_quality/pq_239_influenza_seasonal_10dose_sanofi_pasteur/en/

^3^http://www.interacademies.net/10878/13912.aspx
